# An Intrathermocline Eddy and a tropical cyclone in the Bay of Bengal

**DOI:** 10.1038/srep46218

**Published:** 2017-04-12

**Authors:** Arnold L. Gordon, Emily Shroyer, V. S. N. Murty

**Affiliations:** 1 Lamont-Doherty Earth Observatory of Columbia University, Palisades, NY 10964, USA; 2 College of Earth, Ocean and Atmospheric Sciences, Oregon State University, Corvallis, OR 97331, USA; 3 Council of Scientific and Industrial Research (CSIR)-National Institute of Oceanography Regional Centre, Visakhapatnam, 530017, India

## Abstract

The Bay of Bengal, subjected to monsoonal forcing and tropical cyclones, displays a complex field of ocean eddies. On 5 December 2013 a sub-surface vortex or Intrathermocline Eddy (ITE) composed of water characteristic of the Andaman Sea was observed within the thermocline of the western Bay of Bengal. We propose that the ITE was the product of Tropical Cyclone *Lehar* interaction on 27 November 2013 with a westward propagating surface eddy from the eastern Bay of Bengal. While *Lehar’s* interaction with the ocean initially removes heat from the upper layers of the eddy, air-sea flux is limited as the deeper portions of the eddy was subducted into the stratified thermocline, inhibiting further interaction with the atmosphere. The ITE core from 30 to 150 m is thus isolated from local air-sea fluxes by strong stratification at the mixed layer base, and its periphery is stable to shear instability, suggestive of longevity and the ability to carry water far distances with minimal modification.

The Bay of Bengal (BoB) embayment of the northeastern Indian Ocean, receives an abundance of freshwater by massive river outflow as well as local precipitation, resulting in a warm, low salinity, buoyant surface layer. Freshwater exported from the BoB, with salty water imported from the salty Arabian Sea provides for a quasi-stationary salinity balance at long timescales. Atmospheric forcing is highly seasonal, exposing the BoB to the drier NE monsoon of the boreal winter, and the rainy SW summer monsoon. Tropical cyclones are common from May to December, with peaks in the transitional months of May/June and October/November^1^. The BoB is characterized by an energetic array of westward propagating eddies, which obscure the regionally averaged stratification pattern^2^.

On 27 November Tropical Cyclone (TC) *Lehar* passed over an anticyclonic eddy, evident in a positive sea surface height anomaly (SLA; Fig. 1). Comparison of sea level before and after the passage of *Lehar* reveals a sharp drop from 14 cm to 6 cm from 27 November to 5 December, mainly accomplished by 30 November. The weakened SLA expression of the eddy continued to migrate westward, to be crossed by the Research Vessel (RV) *Revelle* on 5 December 2013. At the time of the ship crossing, the feature was not a surface intensified eddy but rather an Intrathermocline Eddy (ITE). An eddy tracking algorithm^3^ first detects the anticyclone near 13°N, 89°E on 24 August 2013, roughly 550 km from the location of the ship transect of the ITE. After the RV *Revelle* ITE observation the weak positive sea surface anomaly was tracked for another month and ~200 km, until reaching 13.5° N, 82.5° E on 7 January 2014, when it would have encountered the equatorward East Indian Coastal Current. We propose that TC *Lehar* was instrumental in the formation of the subsurface ITE vortex from the surface intensified anticyclonic eddy on 27 November 2013.

## Intrathermocline Eddies

An ITE, a class of subsurface vorticities^4^, have been detected in numerous regions including marginal seas, eastern boundary currents, and within subtropical gyres^5^,^6^,^7^,^8^. ITE at greater depth stemming from interaction of a current with the benthic boundary layer have also been observed^9^. The temperature and salinity properties of ITEs are distinct from those of the surrounding thermocline, suggesting a water type of a remote origin, involving subduction of surface water^10^. An ITE is characterized by relatively homogeneous sub-surface core at the Rossby deformation radius, which in the BoB is ~100 km^11^. A pycnocline dome (cyclonic geostrophic shear) caps the ITE core, while a bowl (anticyclonic geostrophic shear) marks its base. As the pair of opposing geostrophic shears act to compensate each other, the ITE surface expression is weak or absent.

A subsurface vortex fitting the characteristics of an ITE was observed in the western (BoB) during a regional survey by an underway Conductivity Temperature Depth (uCTD), which gathered data within the upper ~200 m from the RV *Revelle* in December 2013 (Fig. 2) as part of the Air-Sea Interactions Regional Initiative (ASIRI) in the northern Indian Ocean^12^,^13^. As ITEs are isolated from the atmosphere by a buoyant surface layer, air-sea fluxes of momentum and heat have a minimal impact on the ITE and so they have the potential of transporting relatively unaltered regional T/S features over great distances. For example, Sarma *et al*.^14^, on examining the water mass stratification (based on hydrographic survey of low spatial resolution) in the eastern Arabian Sea in winter 1982, describe the presence of a subsurface, low salinity feature the 100 to 200 m interval off the west coast of India, at 15°N, 72°E. From temperature and salinity (TS) data they surmise that this feature is derived from the southern Andaman Sea, with subsequent advection around the southern rim of Sri Lanka. The vertical structure and TS properties of the Sarma *et al*. observations are consistent with the ITE observed by the RV *Revelle*, as described below. Babu *et al*.^15^ also describe a doming of the upper thermocline forming an eddy of relatively cold water between 50 and 300 m with a diameter of 200 km, along the western margin BoB centered at 17°40′N, 85°19′E. However, the feature does not have the low salinity subsurface core, and is therefore quite different than the ITE of Andaman Sea water observed by the RV *Revelle*. They conclude that it is a product of baroclinic instability of the southward coastal current with a northward offshore wind induced current.

The ITE core observed by the RV *Revelle* on 5 December 2013 is marked by relatively low salinity of 34.0 to 34.5 g kg^−1^ absolute salinity (S_A_) approximately 1 g kg^−1^ below that of the surrounding water, over the depth range 30 to 150 m. Within the ITE core, there is reduced vertical gradient in salinity, between the 20° and 25 °C, within the 22 to 25 sigma-θ range, revealing a water type distinct from that of its surroundings. Wrapped around the ITE core is salinity maximum near 50–70 m, marking the presence of the Arabian Sea water^2^. The profiles of temperature (Fig. 2c) show that the surrounding fluid in the 40 to 90 m depth interval is 2 °C warmer than the ITE core and 2 °C cooler within the 110 to 150 m interval. The surrounding fluid is denser than the ITE core below 80 m, as the temperature and salinity contrasts compensate each other in the upper 30 m of the ITE. The water type within the ITE core, falling towards the fresher end of the T/S_A_ scatter, contrasts sharply with that of the surrounding water. The lateral edges of the ITE are marked by interleaving structures, while the upper and lower edges of its core lack fine structure.

The width of the ITE core is slightly less than 100 km, as determined by the distance between the locations of the maximum slope of the sigma-θ surfaces in the lower portion of the ITE. As the detection of an ITE by a single section must be considered fortuitous, the maximum diameter of the ITE may very well be greater than 100 km, but this scale is characteristic of ITEs. The density surfaces are depressed downwards at the base of ITE and are uplifted at its crown, marking anticyclonic and cyclonic geostrophic shear, respectively. The ITE Burger (*Bu* = {*N*_*0*_*H/fL*}^2^) and Rossby (*R* = *U/fL*) numbers are order one and roughly equivalent at 0.2. These numbers are similar to those observed previously in other coherent vortices, e.g., in those tracked during the Local Dynamics Experiment and Meddies^16^ though with slightly more variability in previous observations of *Bu* than in *R*. The O(1) values of *Bu* and *R* indicate that both stratification and rotation are important to the dynamics of the ITE.

The velocity field associated with the ITE thermohaline pattern is evident in the RV *Revelle* hull-mounted Doppler sonar systems (Fig. 3a,b). The meridional flow is strongest to the northwest of the ITE, with a pronounced lateral shear apparent in the 50–100 m interval. Across the RV *Revelle* transit the position of near zero current is offset slightly to the northwest of the ITE core with the ITE core exhibiting westward flow of about 0.25 m/sec.

The ADCP section (Fig. 3b) reveals the flow towards the southwest within and south of the ITE core above 175 m, with maximum flow near 50–90 m. As the Rossby number is 0.2 the ITE would be marginally or quasi-geostrophic, none-the-less the ADCP values agree quite well with the geostrophic flow derived from the uCTD data, relative to 200 decibar (Fig. 3c), with the maximum geostrophic current depth, zero geostrophic shear, of ~0.25 to 0.30 m/sec within the 50–80 m depth interval. The surface geostrophic current is in the same direction and about 50% of the subsurface velocity maximum, an indication that the upper cyclonic shear does not fully compensate the deeper anticyclonic shear, resulting in a weak surface anticyclonic flow. The maximum transport swirling around the ITE core is 2.5 to 2.9 Sv (1 Sv = 10^6^ m^3^/s).

Decay of coherent vortices like the ITE can be achieved through diffusion, instability, and/or wave dispersion. The ITE core TS properties not only vary from neighboring stratification, but also in the character of the vertical structure. The fluid surrounding the ITE is characterized by small-scale (~5–10 m) interleaving layers, as highlighted by variations in the Turner angle (Fig. 4), defined as:


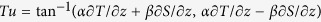


where *α* is the thermal expansion coefficient, *β* is the haline contraction coefficient, and *∂T/∂z* and *∂S/∂z* are the vertical derivatives of temperature and salinity, respectively (vertical increasing upward). The Turner angle^17^ is defined so that regions with 45° < *Tu* < 90° are unstable to salt-fingering and regions with −90° < *Tu* < −45° are unstable to double diffusive convection. Regions with |*Tu*| < 45° are stable to double diffusive process, and regions with |*Tu*| > 90° are statically unstable. The interior of the ITE is well-mixed with both temperature and salinity weakly stabilizing density (ρ_*o*_*β∂S/∂z* ~ −0.002 kg m^−3^ m^−1^ and ρ_o_*α*∂T/∂z ~ −0.01 kg m^−3^ m^−1^). We use the convention that the vertical density gradient, *∂ρ/∂z *=* ρ*_*o*_(*β∂S/∂z* − *α∂T/∂**z***) is negative for a statically stable water column. Note that while we comment on the stability of the ITE to double diffusive processes in a non-sheared system below, the *Tu* is primarily provided as a quantitative measure of temperature and salinity compensation within the ITE and to highlight interleaving layers on the periphery of the ITE.

Interleaving layers on the ITE’s periphery are particularly pronounced on the northwestern side of the ITE as indicated by alternating signs of *Tu* (Fig. 4a). Comparison of individual salinity profiles (Fig. 4c) reveal that small-scale perturbations about the mean can be tracked across isopycnals for distances of over 10 kilometer. Profiles within the ITE core show minimal salinity structure at the upper and lower boundaries of the ITE. In contrast, individual profiles on both the northern and southern edges of the ITE show increased vertical variance with increasing distance from the ITE’s core. Although the proximity of the layers to the ITE might be suggestive of a relationship in isolation, similar layers were observed throughout the >3000 km survey carried out by the RV *Revelle* in locations far removed from the ITE core.

In general, the BoB is quiescent below the mixed layer and is characterized by a complex vertical structure where both salinity and temperature contribute strongly to density (Fig. 4a). Due to these properties, and considering the proximity of interleaving features to the ITE core, it is tempting to consider slow double diffusive processes as a contributor to ITE decay, as has been seen in other ITE-like features^18^. While the fluid between ~50–100 m depth at the edges of the ITE is favorable to double diffusion, within and below the ITE core the water column is not strictly favorable to double diffusion as |*Tu*| < 45°. Moreover, the susceptibility to double diffusive processes (as estimated by considering the amount of water column favorable to double diffusive processes) increases moving away from the ITE’s center. At the level presented here, we cannot support or refute the contribution of double-diffusive layering in ITE decay. However, based on the tendency toward increased layering moving away from the ITE core (Fig. 4c), it seems unlikely that the ITE is strongly affected by double diffusion, at least at the time of the ship survey.

We also evaluate the Richardson number, *Ri *=* N*^*2*^*/S*^*2*^ where *N*^*2 *^=^* *^−*g*ρ_*o*_^−*1*^ *∂*ρ*/∂z* is the square of the Brunt-Väisälä frequency and *S*^*2*^* *=* (∂U/∂z*)^*2*^ is the square of the vertical shear, as a metric for the likelihood of shear instability, with a necessary condition for instability is that *Ri* < 1/4. In the vicinity of the ITE, only unstratified regions within the surface mixed layer have *Ri* < 0.25 (Fig. 4b). The ITE and surrounding fluid are stable to shear instability, based on the density gradient (calculated on scales of 1 m) and vertical shear (calculated on scales of 10 m). Within the core of the ITE *Ri* approaches unity along its base in a narrow band between the 34.3 and the 34.5 g kg^−1^ isohalines, elsewhere it is >5. High values of *Ri* along the stratified edges of the ITE combined with the lack of fine structure at its upper and lower boundaries, suggest that shear-driven mixing is unlikely to be a significant factor in water mass evolution at the time of the ship transect.

## ITE Source Water

The T/S_A_ characteristics within the 20°–25 °C core of the ITE were not observed in any of the other CTD data from the 2013 ASIRI cruises^2^, but are common in the Argo data to the east RV *Revelle* sampling region (Fig. 5). The nearly 6000 Argo profiles within the BoB obtained from 2002 to the end of 2015 (Fig. 5) are inspected for T/S_A_ characteristics found within the ITE (determined by having significant number of data points within the dashed box on T/S_A_ scatter, Fig. 5b). Such profiles are identified by red dots on the map (Fig. 5a). Within the BoB (not including the Andaman Sea) ITE-type water is found in roughly 1.8% of the total 5948 Argo profiles. In the western BoB (i.e., west of the central longitude 88°E) the ITE profiles are only 0.5% of 3229 profiles, whereas in the eastern BoB ITE-type water is found in 3.4% of 2718 profiles. Within the Andaman Sea, ITE-type water is found in 13.4% of 97 profiles. The WOCE section I01 CTD stations data obtained in September 1995^2^ show the preponderance of ITE T/S characteristics within the Andaman Sea.

The prevalence of ITE T/S_A_ water type in the eastern BoB and Andaman Sea, suggests that these regions provide the source waters of the ITE. Sea surface height (SSH) within the BoB is characterized by mesoscale features propagate westward at 0.06 m/s from the Andaman Sea and northeastern BoB^2^, which are generally associated with anticyclonic eddies (high SSH). This is consistent with the estimate that anticyclonic eddy features of Andaman Sea water require about 6 months to cross the BoB^19^. While the source of the T/S_A_ water type found within the ITE core is derived from the east, the significant reduced stratification within the ITE core has not been observed by the Argo profiles, though a few do display somewhat reduced stratification within the thermocline (Fig. 5c). This may simply be a resolution issue due to the limited lateral ITE dimension and the density of the Argo floats.

The distribution of the detected ITE-type water falls within clusters in the eastern BoB (Fig. 5), suggesting that the water types found within the ITE may emanate via the passages connecting the Andaman Sea to the BoB: the Great Channel near 6°N, the Ten Degree Channel and the Preparis South Channel, near 15°N. While analyzing a meridional hydrographic section along 88°E from 4° to near 21°N, Sastry *et al*.^20^ find the zonal geostrophic flow is westward, except in the 11–14°N band, which marks the latitudinal range of Andaman Island, blocking interaction between the BoB and the Andaman Sea. A separate survey revealed water mass characteristics similar to the ITE along the Ten Degree Channel during the southwest monsoon using August 2014 RV *Sindhu Sadana* cruise data. This water mass was traced back to the northern Andaman Sea in the hydrographic section, and acoustic Doppler velocity showed westward currents across the Ten Degree Channel.

## Tropical Cyclone Lehar and the ITE

**Figure 1:**
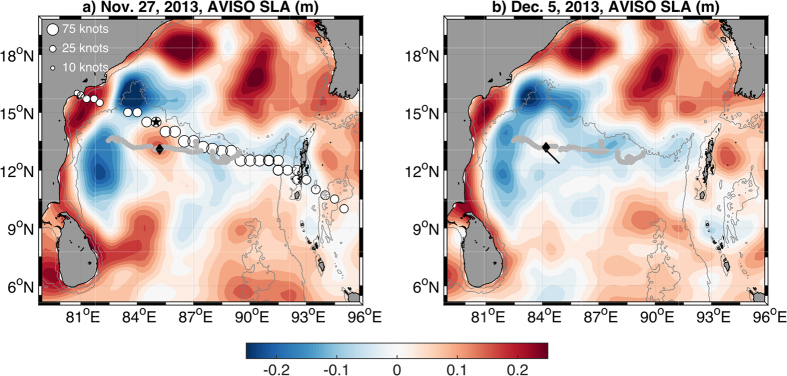
Sea surface height anomaly (contours) from the DT-MSLA “two-sat merged” AVISO product on (**a**) 27 November 2013 and (**b**) 5 December 2013. The World Meteorological Organization’s best track data for TC Lehar is shown every 3 hours from 0300 UTC of 24 November 2013 to 1200 UTC of 28 November 2013 with white markers scaled by the maximum sustained wind speed. The black star indicates the location of TC Lehar at 1200 UTC on 27 November 2013. Grey markers show the anticyclone trajectory^3^. The black marker shows the centroid of the eddy on the day of the SLA fields. The black line on the 5 December 2013 panel shows the ship transect through the ITE. The map was produced using Netcdf toolbox of Mathworks Matlab, https://www.mathworks.com/products/matlab/. The Sea surface height anomaly data are from Aviso: The Ssalto/Duacs altimeter products were produced and distributed by the Copernicus Marine and Environment Monitoring Service (CMEMS) (http://www.marine.copernicus.eu): http://www.aviso.altimetry.fr/en/data/products/sea-surface-height-products/global; which is accessible from http://wombat.coas.oregonstate.edu/eddies/The Tropical cyclone data are from: The Climate Data Guide: IBTrACS: Tropical cyclone best track data^28^, which is retrieved from: https://climatedataguide.ucar.edu/climate-data/ibtracs-tropical-cyclone-best-track-data.

**Figure 2:**
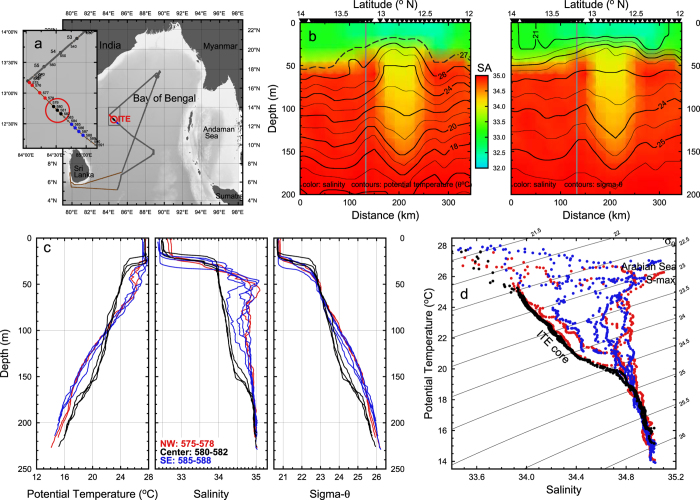
Temperature and salinity stratification of the ITE. (**a**) Track of the RV Revelle ASIRI leg 2, 27 November to 13 December 2013. The insert map details the position of the uCTD stations. The core of the ITE (red circle) was sampled on 5 December 2013, uCTD stations 580–582. (**b**) The uCTD section crossing the ITE color-coded by absolute salinity, with contours of potential temperature (left panel) and sigma-θ (right panel). The gray vertical line marks the course change shown on the map. (**c**) The potential temperature, salinity and sigma-θ profiles within the ITE core and to its northwest and southeast. (**d**) The T/S of the ITE core and of the waters surrounding the ITE. The salinity maximum (S-max) of the Arabian Sea water and of the ITE core are marked. The map was produced from GMT 4.5.2 http://gmt.soest.hawaii.edu/ with the sea floor bathymetry from Sandwell/Smith 18.1, http://topex.ucsd.edu/marine_topo/.

**Figure 3:**
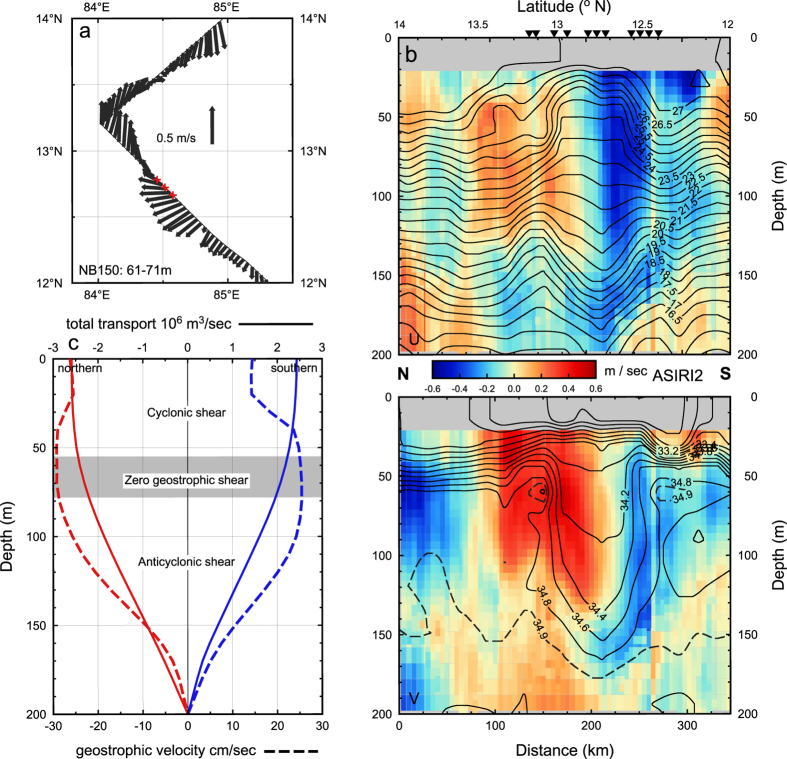
ITE circulation- (**a**) Velocity associated with the ITE from the RV Revelle ADCP within the depth interval 61–71. (**b**) The ADCP velocity section, the zonal component (+ is eastward) is the upper panel and meridional component (+ is northward) in the lower panel. The section is constructed from both the 75 and 150 kHz data: 75 kHz 38 m and deeper; the 150 kHz records the 21–38 m interval. Isotherms are shown on the u-component; salinity on the v-component. (**c**) The geostrophic velocity (dashed lines) and transport (solid lines) relative to 200 decibars, for stations to the northwest (red) and to the southeast (blue) of the ITE core. The Map was produced with GMT 4.5.2, http://gmt.soest.hawaii.edu/.

**Figure 4:**
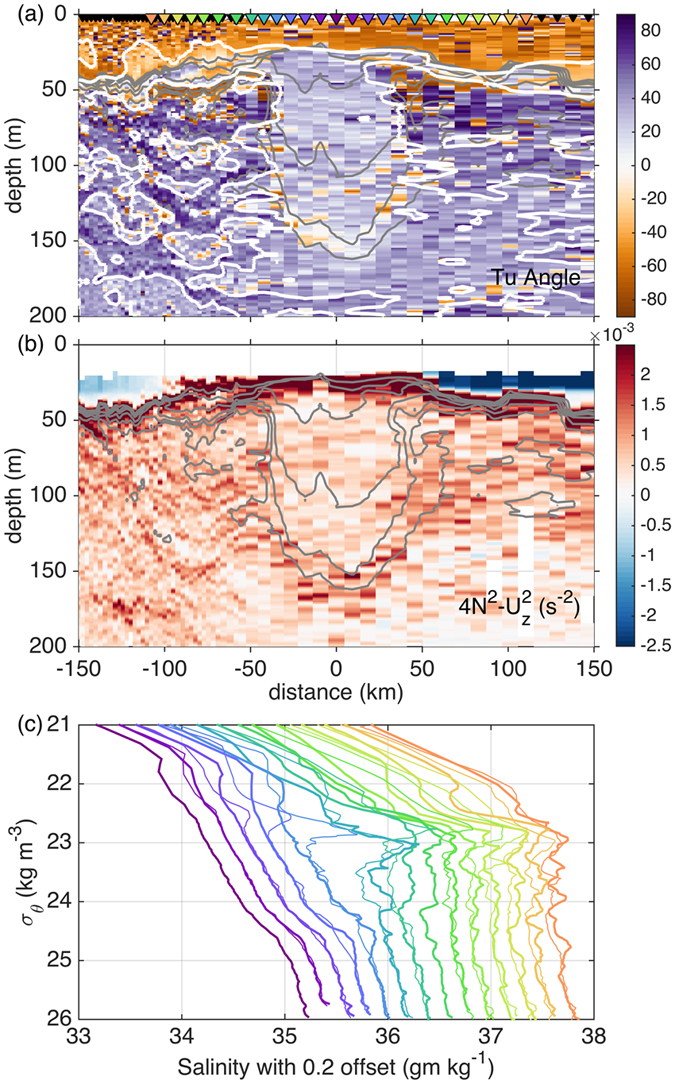
Distance-depth section plots of the stratification of the ITE. (**a**) Vertical Turner angle (colors) with |Tu| = 45° contoured in white and (**b**) 4N^2^ - S^2^ in the vicinity of the ITE. N is the Brunt-Väisälä frequency; S^2^ = (*∂*U/*∂*z)^2^. When 4N^2^ - S^2^ is zero the stratification is sufficient to suppress shear instability. Isohalines are contoured in grey in increments of 0.25 g kg^−1^. (**c**) Profiles of salinity offset by 0.2 g kg^−1^ moving outward from the ITE centerline. Profile locations are indicated by colored markers in panel a; profiles from the northern (southern) side of the ITE are shown with thick (thin) lines.

**Figure 5:**
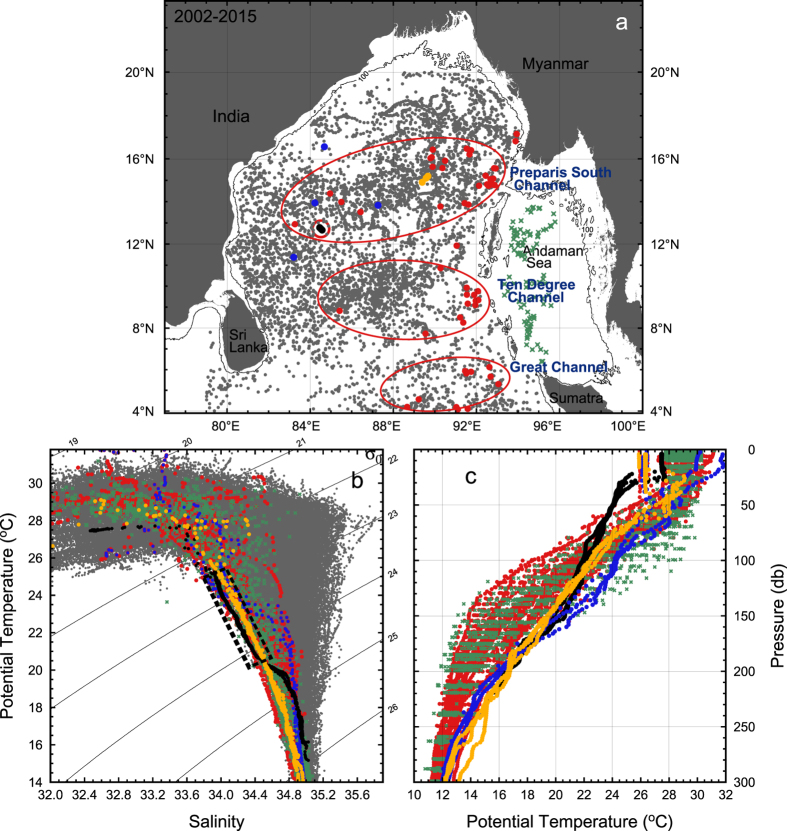
Argo profiles collected through the end of 2015 in the BoB and Andaman Sea. (**a**) Map of Argo profiles, with red designating T/S_A_ characteristics that have substantial data points within the T/S_A_ values of the ITE core. Circled are 3 grouping of Argo profiles with ITE T/S_A_ characteristics, which can be related to the passages connecting the Andaman Sea to the BoB; (**b**) The T/S_A_ scatter of the ‘red dot’ Argo profiles that contain T/S_A_ values similar to the RV Revelle ITE within the black dashed box. T/S_A_ of Argo profiles in the Andaman Sea are shown as green + symbols; and (**c**) temperature profiles of the Argo profiles that display T/S_A_ similar to the ITE. Black symbols are from the core of the ITE as observed by the RV Revelle. A few Argo floats detect weaken thermocline: yellow symbols are from Argo profiles at ~15°N, 89.5°E obtained in January 2013; the blue symbols are from various Argo floats from early 2015. The map was produced using, GMT 4.5.2 http://gmt.soest.hawaii.edu/ with the sea floor bathymetry from Sandwell/Smith 18.1 http://topex.ucsd.edu/marine_topo/. The Argo profile data are from http://www.nodc.noaa.gov/argo/.

While the low salinity water masses characteristic of the ITE are clearly derived from the Andaman Sea, the issue of when and how these water masses were incorporated into the ITE dynamical structure is not resolved. We speculate that at some point a westward propagating anticyclonic eddy of Andaman Sea water was transformed into the observed ITE structure. The likely timing of this event might have been slightly more than a week before the ITE was observed from the RV *Revelle*, upon passage of TC *Lehar* (Fig. 1).

Although typically cooling and reduced SST is associated with the passage of a tropical cyclone, there is only weak TC-induced cooling in the northern BoB during the post summer monsoon, due to salinity-stratified mixed layers and the associated barrier layer 21,22. The BoB is often characterized by subsurface warm layers^23^ that can even warm the sea surface through subsurface mixing during intense forcing as observed in the eastern BoB during the TC *Hudhud*^24^. These factors may explain the weak SST cooling, implied by comparison of mixed layer and ITE temperature. Ekman upwelling induced by TC *Lehar* would shallow the upper thermocline whereas the lower thermocline of the anticyclonic eddy would not, or to a lesser degree, be affected by the TC forcing. Straining by Ekman pumping combined with presumably elevated vertical mixing by winds would produce the low stratification observed within the ITE core on 5 December.

The interaction of strong TC with cyclonic ocean eddies (COE) have been studied in the North Pacific Ocean using satellite derived SST and SSH^25^, and within a model^26^. The observational study finds that the pre-existing COE intensified upon passage of a strong TC, and a few new COE were generated. Based on the observations reported here we propose that an anticyclonic ocean eddy would have the oppose reaction: they would weaken. We add that while their surface expression weakens their subsurface expression remains, forming an ITE. The model based study^26^ also finds that the COE were enhanced. They point out “However, the other eddy type (anticyclonic), size, and strength were not accounted for this study”. It is expected that the response of anticyclonic ocean eddy (AOE) would be opposite that that of the COE, as supported by the Parallel Ocean Program model using a spatially uniform, but rotating wind field^27^ (which may be quite appropriate for a passing TC).

## Conclusion

An ITE was observed on 5 December 2013 in the western BoB. While the water mass origin within the ITE core is derived from the east, most likely from the Andaman Sea, the dynamic structure of the ITE is not necessarily a product of that region, and instead may be of local origin. SSHA reveals the presence of a westward propagating anticyclonic eddy (Fig. 1) that dramatically attenuated, with the SSHA signal more than halving, initiated with the passage of TC *Lehar* on 27 November, prior to continuing its westward drift (Fig. 1). The alteration of the anticyclonic eddy upon passage of TC *Lehar* on 27 November is a coincidence too great not to suspect that the TC *Lehar* played a pivotal role in transforming the anticyclonic eddy into the ITE.

Our observations illustrate rapid transformation of a surface-intensified anticyclone into subsurface ITE after passage of a TC. As the submergence of an anticyclonic eddy may feedback to the TC evolution and that ITE core water can carry water properties, protected from the local air-sea fluxes, over great distances from their origin, this process may have important larger-scale regional significance to both the atmosphere and ocean systems.

The weak sea surface signatures of ITEs present a challenge to accurately diagnosing subsurface structure and accounting for water mass transport from remotely sensed surface data, which alone would suggest simple decay of the anticyclonic eddy rather than conversion to an ITE. The possibility of the transformation of an anticyclonic eddy into a ITE feature in response to a TC is a worthy target for future research. We advocate specifically designed targeted field activity, that can directly observe the response of an AOE to an encounter with a TC, along with a model based study of TC interaction with an AOE.

## Additional Information

**How to cite this article**: Gordon, A. L. *et al*. An Intrathermocline Eddy and a tropical cyclone in the Bay of Bengal. *Sci. Rep.* **7**, 46218; doi: 10.1038/srep46218 (2017).

**Publisher's note:** Springer Nature remains neutral with regard to jurisdictional claims in published maps and institutional affiliations.
